# Cell-Electrospinning and Its Application for Tissue Engineering

**DOI:** 10.3390/ijms20246208

**Published:** 2019-12-09

**Authors:** Jiyoung Hong, Miji Yeo, Gi Hoon Yang, GeunHyung Kim

**Affiliations:** Department of Biomechatronic Engineering, Sungkyunkwan University (SKKU), Suwon-si, Gyeonggi-do 16419, Korea; kongjenice@gmail.com (J.H.); yeomiji93@gmail.com (M.Y.); gthang51@skku.edu (G.H.Y.)

**Keywords:** cell-electrospinning, micro/nano structure, cell-laden scaffold, tissue engineering

## Abstract

Electrospinning has gained great interest in the field of regenerative medicine, due to its fabrication of a native extracellular matrix-mimicking environment. The micro/nanofibers generated through this process provide cell-friendly surroundings which promote cellular activities. Despite these benefits of electrospinning, a process was introduced to overcome the limitations of electrospinning. Cell-electrospinning is based on the basic process of electrospinning for producing viable cells encapsulated in the micro/nanofibers. In this review, the process of cell-electrospinning and the materials used in this process will be discussed. This review will also discuss the applications of cell-electrospun structures in tissue engineering. Finally, the advantages, limitations, and future perspectives will be discussed.

## 1. Introduction

Tissue engineering has achieved noteworthy advancements in regenerating human organs and tissues ever since it was introduced. Tissue engineering focusses on restoring or improving the function of defective tissues by developing biological substitutes, comprising scaffolds, cells, and biofactors [1]. Therefore, the scaffolds are expected to provide a cell-friendly environment from micro- to nanoscale to guide cells into the targeted tissue or organ and enable them to mature. For this reason, various methods have been investigated to design bioscaffolds that simulate the environment of the native tissue.

**Figure 1:**
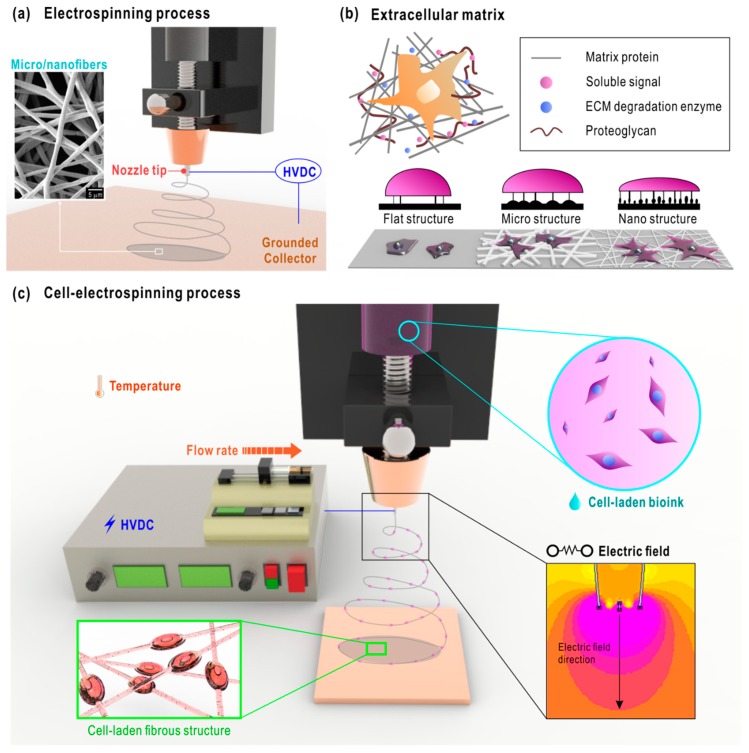
(**a**) Electrospinning process with the basic components. (**b**) Extracellular matrix (ECM) structure and cell–cell interconnectivity on a flat, micro, and nano structure. (**c**) Cell-electrospinning process with the processing parameters.

Electrospinning (ES) is one well-known method of fabricating scaffolds that comprise micro/nanofibers. The early concept of electrospinning was proposed in the 1930s by Anton Formhals. Since its introduction, it has been used in various fields, such as aerospace applications, agriculture, filtration, and textile [2,3,4,5,6]. ES is based on the use of electrical forces to produce fibers with sizes ranging from micro- to nanometers. An electrospinning process basically requires three components, namely a nozzle tip attached to a high voltage direct current (HVDC) source, a flow rate controller, and a grounded collector, as illustrated in Figure 1a [7]. When the electric field is applied between the nozzle tip and the grounded collector, a microsphere is formed at the end of the nozzle. As the strength of the electric field increases, the microsphere at the tip elongates forming a conical shape called the Taylor cone. The electrostatic force within the Taylor cone becomes greater than the surface tension, thereby generating a liquid jet from the cone. The jet undergoes bending instabilities, causing the whipping of fibers, resulting in a randomly oriented fibrous mat [8]. These micro/nano-sized fibers provide a large surface area-to-volume ratio, which can enhance cellular activities, such as cell attachment, proliferation, and differentiation [9]. Moreover, these electrospun scaffolds simulate the native structure of an extracellular matrix (ECM), which demonstrates a vital role in cell functions, such as cell survival, polarity, proliferation, migration and differentiation [10,11]. ECM also plays an important role in cell–ECM interaction, transducing extracellular information into intracellular events (Figure 1b) [12,13].

Consequently, electrospun fibers are widely used in biomedical applications to regenerate various tissues. For example, Chong et al. used a polycaprolactone (PCL)/gelatin blend to develop an artificial skin layer using the ES method. In this study, PCL provided mechanical stability, while gelatin was included to increase the biocompatibility of the electrospun scaffolds [14]. In another study, a collagen/chitosan/poly(L-lactic acid-co-ε-caprolactone) (PLLA-CL) blend was electrospun to fabricate a vascular graft. Though collagen and chitosan provided superior biocompatibility, they demonstrated poor mechanical properties, and therefore, PLLA-CL was employed [15]. Some tissues, such as muscles, nerves, and tendons, are composed of highly aligned structures. Therefore, many studies have been conducted to generate aligned fibers. Aviss et al. used a rotating mandrel as the grounded collector to achieve aligned poly(lactide-co-glycolide) fibers for skeletal muscle regeneration. The diameter of the fiber could be adjusted by varying the rotational speed of the mandrel orientation [16]. Another method involved depositing PCL fibers in the gap that separates two grounded poles. The fibers were aligned by moving back and forth between the poles, resulting in an aligned PCL bundle. This 3D fibrous bundle was fabricated for nerve regeneration [17]. ES has gained significant interest in biomedical applications due to the aforementioned benefits. Nevertheless, it demonstrates some limitations, such as usage of toxic solvents, poor cell infiltration, and inhomogeneous cell-distribution.

To overcome these limitations, a novel method, called cell-electrospinning (C-ES), was introduced. C-ES is an electrospinning-based technique that generates fibers with living cells embedded within (Figure 1(c)) [18]. The major difference between conventional ES and C-ES involves the use of viable cells. To elaborate, C-ES was firstly introduced by Jayasinghe et al. in 2006, to demonstrate the feasibility of developing active biological scaffolds using a modified ES process [18]. In this study, astrocytoma (1321N1) cells were embedded in cell suspension, which was supplied to the inner needle of the core–shell nozzle. The polydimethylsiloxane solution was supplied to the outer needle to enhance the mechanical properties of the cell-laden fibers. The cell viability was measured as 67.6 ± 1.9%, which was close to the genuine viability (~70%) of astrocytoma cells. The metabolic activities and cell proliferation were maintained for six days, and the compatibility of C-ES was established. This concept was then expanded by using various types of cells (adipose stem cells, osteoblasts, cardiac myocytes, and neuroblastoma) and various biocompatible materials (polyvinyl alcohol (PVA), alginate, and Matrigel) [19,20,21,22]. As the possibility of the C-ES process has been revealed, a comprehensive investigation is required to apply this process for realistic biomedical applications.

In this mini-review, we extensively investigate the C-ES process. Firstly, the processing of C-ES is introduced and the parameters affecting the process are discussed. The biomaterials used in the C-ES process are also described. Secondly, we introduce several applications of the C-ES process for biomedical purposes. Finally, the advantages, limitations, and future perspectives of C-ES are presented.

## 2. Processing of C-ES

Several critical conditions, such as the viscosity of the bioink, the applied electric field, the feeding rate of the bioink, the distance between the tip of the nozzle to collector, and environmental factors, influence the C-ES process.

### 2.1. Material Parameters

The viscosity and surface tension of the printing solution are important to the development of cell-loaded fibers using C-ES. The viscosity of the bioink not only affects the cell-embedding efficiency, but also the printability [23]. Generally, low-viscosity bioinks are advantageous, as higher viscosities result in greater shear stress which can negatively affect the cells [24]. Kim et al. observed a significant decrease in cell viability (<80%) when the collagen content in the bioink was greater than 7 wt% [25], while a high cell viability (of 93%) was achieved when the collagen weight percent was less than 5. However, bioink with a very low viscosity may cause the spraying of solution droplets instead of generating fibers [26,27].

In the conventional ES process, the surface tension is controlled by changing the polymer/solvent ratio of the solution [27]. In a study by Lee et al., the surface tension was dependent on the volume ratio of the two solvents (methylene chloride and *N*,*N*-dimethylformamide) used for PCL electrospinning [28]. In general, solvents may damage the viable cells, rendering them unsuitable for C-ES [29]. Therefore, selecting the polymers for proper solution conductivity is critical.

### 2.2. Process Parameters

One of the most important parameters in ES-based techniques is electric field, without which electrospinning is impossible [8]. As C-ES involves the fabrication of cell-embedded fibers, the strength of the electric field is of very significant. In ES, the use of the electric field is considered as a parameter affecting the generation of fibers, while cell viability is also considered during the E-CS process. Strong electric fields may cause low cell viability [21,30]. For instance, high cell viability (90%) was achieved when the electric field was in the range of 0.05–0.075 –kV/mm. However, a significant drop in cell viability was observed when the strength of the electric field was increased [30]. In contrast, weak electric fields may result in inappropriate fiber formation [21,30]. In another study, the application of a low electric field (0.1 kV/mm) resulted in high cell viability (90%); however, the microfibers generated were not well-developed [21]. Therefore, the distance between the tip of the nozzle to the collector—which influences the electric field—is also an important variable [31]. Another parameter that affects the C-ES process is the flow rate of the solution. The flow rate is vital, not only for fiber formation, but also to achieve a high cell viability. It is also closely related to shear stress, and therefore, should be determined depending on the material.

In addition, some environmental factors, such as temperature and humidity, may influence the C-ES process. Temperature, especially, may affect the cell viability and the rheological properties of the bioink. In terms of cell viability, the temperature of the printing environment may affect the cells during the holding time [32]. Moreover, it is prominent that the rheological properties are affected by temperature [33]. It was reported that the synergistic effects of decreased viscosity and decreased surface tension affected fiber morphology. In some cases, the temperature was also used to thermally crosslink the material [34]. However, the C-ES process must be conducted in controlled temperatures, so that the cells are not damaged. Therefore, temperature must be carefully regulated during this process. Humidity is another critical factor affecting fiber formation. A high humidity results in beaded fibers, because it enables the initial jet to elongate longer [35]. When the initial jet is longer, the charge on the surface of the jet declines due to an increased surface area, and this phenomenon leads to an unstable jet, causing capillary instability. When electrospinning is conducted under high humidity conditions, the beads are formed in between the thin fiber segments [35,36]. Therefore, to achieve C-ES, the cell-embedded solution must be precisely electrospun under the appropriate room humidity to fabricate bead-free fibers.

## 3. Biomaterials for Cell Electrospinning

In general, biomaterials are classified into two groups: naturally and synthetically derived polymers (Table 1). For achieving micro/nanofibers, synthetic polymers have been widely used in ES by selecting the appropriate solvents that can deliver the required viscosity, surface tension, and conductivity [37]. Owing to their high processability, many synthetic polymers, such as polymethylmethacrylate, polycaprolactone (PCL), and polylactic acid, were developed into fibrous structures through ES. However, the solvents used for ES—tetrahydrofuran, acetone, and chloroform—are usually toxic to cells. Even though removing these toxic solvents is necessary for cell culture, it is not appropriate for achieving biofabrication using living cells. Nam et al. demonstrated that solvent retention in the electrospun scaffolds composed of PCL, gelatin, and PCL-gelatin blend [38]. Canbolat et al. also showed that the residual solvent in the electrospun scaffolds affected the cell viability, and cleansing of the scaffolds only improved the cell viability by 5–10% [39]. Therefore, natural polymers are used in the C-ES process, owing to their cell-friendliness. Various natural polymers, such as alginate, collagen, and cellulose, are utilized for ES. Although these are biocompatible and hydrophilic, they reveal some drawbacks in electrospinnability, due to weak molecular chain entanglement or a repulsive force among ions [40,41]. To overcome these limitations, the C-ES process employs a core–shell nozzle or a mixture of synthetic and natural polymers. Table 2 lists the advantages and disadvantages of ES and C-ES processes.

### 3.1. Synthetic Polymers

Two of the synthetic polymers used in C-ES are poly(dimethylsiloxane) (PDMS) and polyvinyl alcohol (PVA) [18]. One of the very first C-ES processes used PDMS to encapsulate the cells in the fibers [18]. The PDMS was fed through to the outer needle at a 10^−8^ m^3^/s flow rate, which was utilized to provide mechanical strength. Meanwhile, the cell-laden biosuspension was fed through to the inner needle at 10^−8^ m^3^/s, and the finest cell-laden thread was fabricated using a 0.09 kV/mm electric field. The measured astrocytoma cell viability (67.6 ± 1.9%) was similar to the control astrocytoma cells (70.6 ± 5.0%) on a 2D culture plate. In another study, adipose stem cells (ASCs) were embedded in the microthreads composed of PVA [22]. This study will be discussed thoroughly later in this review.

### 3.2. Natural Polymers

Naturally derived biomaterials have shown many advantages over synthetic polymers as a material for bioink. Natural polymers provide bioactive cues which promote cellular functions. So, few natural polymers or blends of them have been used in order to replace synthetic materials. For instance, alginate with a poly(ethylene oxide) (PEO) and lecithin blend was used for bone regeneration. MG63, osteoblast-like cells, were used for this process. [21]. As a result, an electric field strength of 0.16 kV/mm showed the greatest cell viability of over 80%, with fibers ranging from 5 to 17 μm. Similarly, Yeo et al. also used alginate as the bioink material, together with PEO and CaCl_2_, as the crosslinker for skeletal muscle regeneration [30]. Here, the optimized electric field was 0.075 kV/mm and the cell (C2C12) viability was greater than 90%. As another C-ES material, a modified, Matrigel-rich collagen biopolymer was used for a different muscle tissue regeneration. Primary cardiomyocytes contained in the collagen-based bioink were electrospun by applying a voltage of 230 V and current of 50 mA [30]. No significant alterations were observed in the cell viability or function of the myocytes after the C-ES process. Gelatin, which is a collagen derivative, is also widely used in the tissue engineering field, since it has similar properties to collagen: great biodegradability and biocompatibility. However, its poor fiber-forming ability restricts its use in C-ES [56].

## 4. Application in Regenerative Medicine Using Different Cells

Since various tissues require tailored micro-/nano-environments for tissue regeneration, C-ES has been applied using various biomaterials and different strategies to build cell-laden fibers (Table 3). Briefly, in this section, the attempts at regenerating different types of tissues using C-ES will be introduced.

### 4.1. Bone Cells

**Figure 2:**
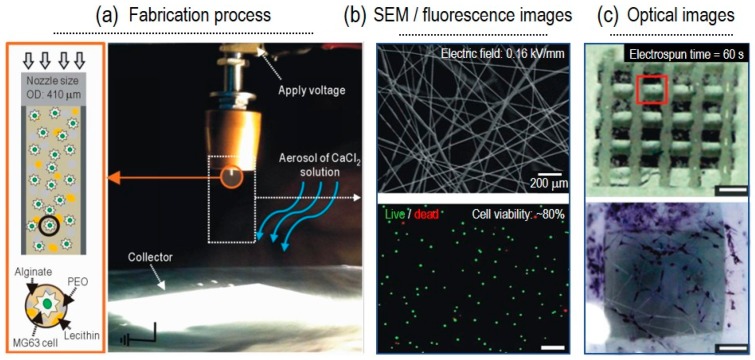
(**a**) Schematic and optical image for fabrication process. (**b**) SEM/fluorescence (live/dead) images of fabricated cell (MG63)-laden electrospun fibers. (**c**) Optical images of alkaline phosphatase (ALP) stained cells. Figure adapted with permission from [21]. Copyright 2015 Elsevier.

As bone defects, such as infections, osteonecrosis, and cartilage problems, have been increasing, the importance of bone tissue regeneration has been increasing. As autologous bone graft demonstrates some limitations in terms of donor site morbidity and graft volume, allogenic bone regeneration medicine has received considerable attention. Even though 3D printing has been widely used to fabricate bone regenerative scaffold, it does not guarantee a high surface-area-volume ratio and high resolution, which can enable the fabrication of an ECM-like structure. Yeo et al. combined 3D printing and C-ES to complement a 3D structure with high mechanical strength and an ECM-like structure for bone regeneration [21]. MG63 human osteosarcoma cells (2 × 10^5^ cells/mL) were encapsulated in alginate/PEO/lecithin solution and electrospun with a 0.16–kV/mm electric field and a 0.5–mL/h flow rate (Figure 2). The electrospun cells revealed a cell viability of over 80%, and their osteogenic differentiation was confirmed using alkaline phosphatase staining after 10 days of culture. Consequently, it could be established that C-ES enhanced bone regeneration by supplementing 3D printing with hierarchically designed, 3D cell-laden micro/nanofibrous structures.

### 4.2. Muscle Cells

**Figure 3:**
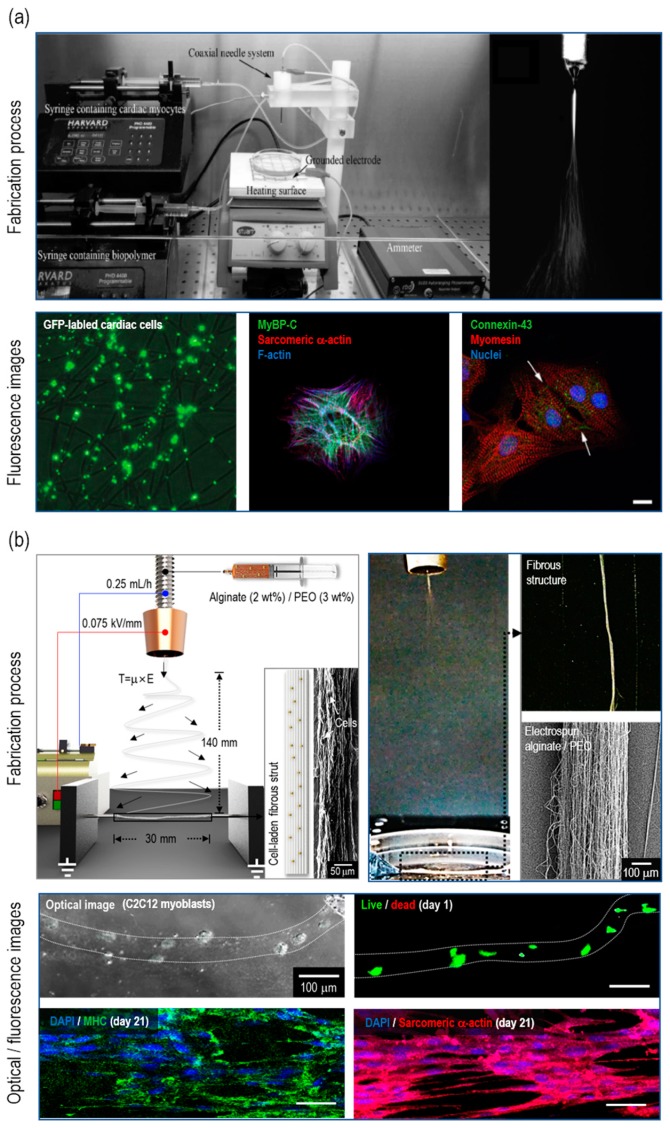
(**a**) Optical images of the fabrication process for electrospun cardiac patch. Fluorescence images of GFP-labeled cardiac cells (left), MyBP-C/sarcomeric α-actin/F-actin stained cardiac cell (middle), and connexin-43/myomesin/nuclei stained cardiac cell (right). Figure adapted with permission from [57]. Copyright 2014 The Royal Society of Chemistry. (**b**) Schematic/optical/SEM images of electrospinning process for generating aligned micro/nanofibers. Optical/fluorescence images of electrospun C2C12 myoblast cells-laden micro/nanofibers. Figure adapted with permission from [30]. Copyright 2018 WILEY-VCH Verlag GmbH & Co. KGaA, Weinheim.

Human muscle plays a prominent role in the whole body, as it comprises 45% of body mass and regulates stability and metabolism. The muscle tissue is made up of muscle cells which can be divided into three types: cardiac, skeletal, and smooth [63,64,65]. In a study by Ehler et al., primary neonatal rat cardiomyocytes were used to fabricate 3D cardiac patches for repairing ageing and/or damaged cardiac tissues [57]. The myocytes-laden Matrigel-rich collagen biopolymer was electrospun with a 3.6 mL/h flow rate and a 0.4 –kV/mm electric field (Figure 3a). The cell viability after C-ES was similar to that of the cells on the culture dish, which was approximately 80%. Then, the immunofluorescence staining of myosin binding protein C, sarcomeric α-actinin, connexin-43, and myosin revealed that C-ES supported the integrity of cardiac myocytes. The skeletal muscles demonstrate a long multinucleated structure and tissue layers arranged in parallel [66,67]. Aligned physical/biochemical cues have been generally known to accelerate muscle regeneration. In this regard, the aligned cell-laden micro/nanofibrous structure was assessed to observe cellular activities in comparison with 3D cell-printed structure [30]. The same bioink, alginate/PEO embedded with C2C12 cells (mouse myoblast cell line) (5 × 10^6^ cells/mL), was used for both the C-ES and cell printing processes. For C-ES, an electric field of 0.075 –kV/mm strength, and a flow rate of 0.25 mL/h, were employed (Figure 3b). Cell morphology was captured at day 7, and it was observed that the cells on the micro/nanofibers aligned and elongated 3-fold compared with those on the printed structure. In addition, the formation of myosin heavy chains and sarcomeric α-actinin was significantly increased for the C-ES scaffold (Figure 3b). In short, muscle tissue engineering using C-ES illustrates not only compatibility with diverse types of muscle cells, but also controllability in micro-/nano-topological cues.

### 4.3. Other Cells

**Figure 4:**
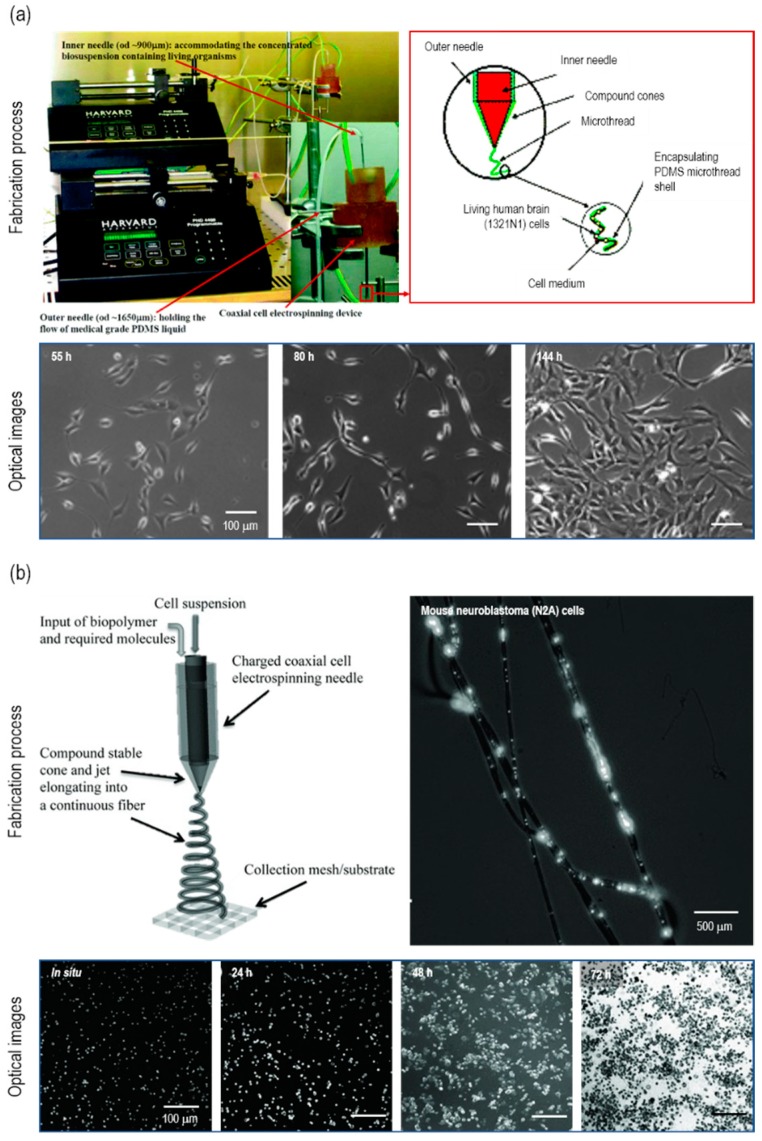
(**a**) Optical/schematic image of the fabrication process for cell electrospinning using 1321N1 human astrocytes. Optical images of the 1321N1 cells cultured for 55 h (left), 80 h (middle), and 144 h (right). Figure adapted with permission from [18]. Copyright 2006 American Chemical Society. (**b**) Schematic/optical image of cell electrospinning using mouse neuroblastoma (N2A) cells. Optical images of electrospun N2A cells in situ and after 24 h, 48 h, and 72 h. Figure adapted with permission from [20]. Copyright 2013 WILEY-VCH Verlag GmbH & Co. KGaA, Weinheim.

Townsend-Nicholson et al. developed a C-ES process with a coaxial nozzle to dispense cell-laden media in core and poly(dimethyl siloxane) (PDMS) in shells [18]. 1321N1-immortalized human astrocytes (1 × 10^6^ cells/mL) were suspended in media and electrospun using a 0.36-mL/h flow rate and a 0.09-kV/mm electric field (Figure 4a). The cell viability was 67.6 ± 1.9% and 70.6% ± 5.0% for the electrospun 1321N1 cells and the control cells cultured in a Petri dish, respectively. The electrospun cells were as viable as the control cells, and these results correspond to the typical 1321N1 cell viability (65~75%). The electrospun cells proliferated through 9 days of culture, which demonstrates that 1321N1 cells were capable of maintaining metabolic activities after C-ES. Furthermore, mouse neuroblastoma N2A cells (5 × 10^5^ cells/mL) were examined to compare control (2D culturing) and two experimental groups (C-ES and aerodynamically-assisted bio-threading (AABT)) [20]. To encapsulate N2A cells, a modified matrigel with a high concentration of laminin was used for both C-ES and AABT (Figure 4b). The C-ES scaffold revealed a cell viability from 60% to 85% throughout days 1 to 3, in which the result was similar to the AABT scaffold (Figure 4b). For in vivo tests in mice, the proliferation rate among the three groups was also similar, which reveals the adequate biocompatibility of C-ES.

### 4.4. Stem Cells

**Figure 5:**
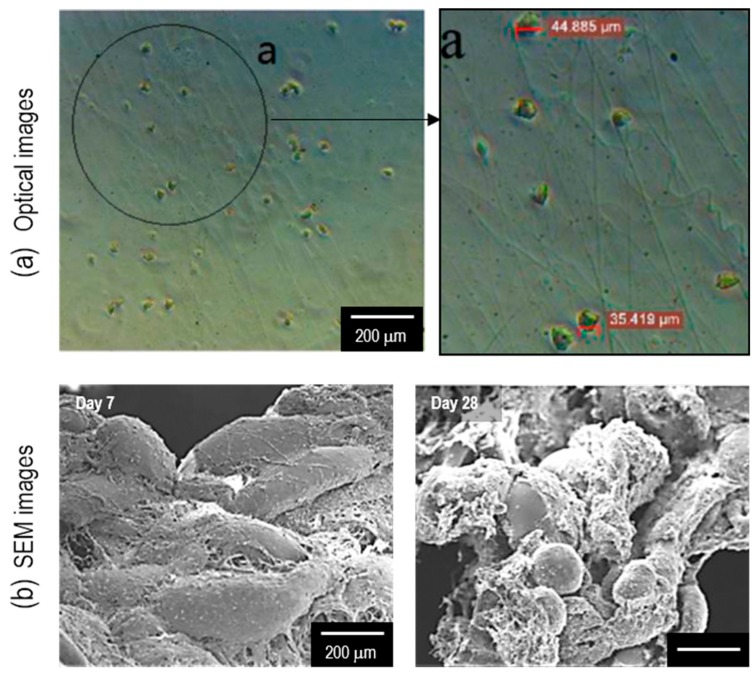
(**a**) Optical images of electrospun adipose stem cells (ASCs). (**b**) SEM images of ASCs after 7 days and 28 days of culture. Figure adapted with permission from [22]. Copyright 2015 Elsevier.

Stem cells are generally more vulnerable to external stimuli, and therefore, the fabrication of viable ECM-like microenvironments is essential for regenerating tissues using stem cells. To elaborate, Chen et al. developed a bioactive membrane through C-ES using ASCs [22]. In the work, they used 8.8% PVA (M_w_ = 2000) mixed with ASCs (1 × 10^7^ cells/mL). Then, the electrospinning solution was flowed at 9.6-mL/h and applied with 0.8-kV/mm. The cellular activities of ASCs on electrospun fibers were assessed using the ASCs on a cell culture dish. After 28 days of cell culture, the ASCs on electrospun fibers managed to survive and proliferate by 133%, while those on the culture dish decreased by 18% from day 1 (Figure 5). This experiment established that ASCs were compatible with C-ES and a fibrous architecture was preferred to sustain cellular activities.

## 5. Concluding Remarks

### 5.1. Advantages of C-ES

Cell-electrospinning has been widely used not only in the bio-engineering field, but also in various other fields of study. This technology enables the fabrication of nanoscale fibers using a simple process. In contrast to other methodologies (e.g., cell-printing) that demonstrate limitations in fabricating micro-sized fibers, C-ES produces nano-sized cell-electrospun fibers that provide higher resolutions and better ECM-like structures. In addition, the cell-electrospun fibers demonstrate the synergistic effect of nanoscale patterning by guiding cells along the fibers, rather than just simulating the size of the structure. Also, the cell-electrospun fibers in nanoscale enable an efficient and fast exchange of nutrients and oxygen. Finally, the cell-electrospun fibers provide better cell-to-cell interaction than the cell-embedded bulk structure. Overall, we anticipate that C-ES can be used for a wide range of clinical applications in the area of regenerative medicine.

### 5.2. Limitations of C-ES

Although C-ES has been recognized as a leading biotechnology, it demonstrates some limitations. As the process of C-ES uses hydrogels to encapsulate cells in the fibers, the mechanical strength of the resulting structure may be low. Furthermore, the restrictions encountered by C-ES, with respect to the development of 3D structures, are still not addressed, which is also a limitation encountered in the conventional ES process. Another downside of C-ES is the low controllability in terms of cell density compared to 3D cell-printing techniques. Furthermore, achieving fiber deposition on precise location is challenging due to the whipping phenomenon that occurs during the generation of fibers.

### 5.3. Future Perspectives

C-ES, which is an emerging biomedical technique, was elaborated upon in this study, and its applications, advantages, and limitations were discussed. The capabilities of C-ES include embedding the living cells directly in nanoscale fibers and guiding the cells to grow in the fiber direction. These characteristics enable cellular activities, by providing physical cues and enlarging cell-to-cell/matrix interactions. However, several challenges still remain. Therefore, further studies are required to improve the mechanical properties—as well as cell density—and overcome the restrictions with respect to the development into 3D structures. To overcome the limitations and enhance the mechanical properties and shape-ability, modified techniques, such as cell-electrospinning combined with 3D printing, have emerged. In addition, developing a cell-electrospun structure with biological functional factors has been challenging. Therefore, a platform for co-culturing, or multiple cell culturing, should be actualized to simulate the physiological functions of complex tissue (i.e., vascularized muscle structure or skin layers comprising multiple cell types such as keratinocytes and fibroblasts). If these drawbacks are addressed, cell-embedded electrospun nanofibers demonstrate significant potential in the application of tissue engineering scaffolds.

## Figures and Tables

**Table 1:** Description of materials used for cell-electrospinning classified into natural and synthetic polymers.

Biomaterial		Description	Advantages	Disadvantages
**Natural polymer**	Collagen [42,43,44]	Most abundant protein in mammals Main protein of extracellular matrix (ECM) Mostly found in tendons, ligaments, bone and skin tissue	Highly biocompatible and biodegradable Relatively nonimmunogenic	Poor mechanical strength Expensive
	Gelatin [45,46]	Derived from collagen Similar properties with collagen	Biocompatible and biodegradable Relatively low cost	Poor mechanical strength
	Alginate [47,48,49]	Derived from cell walls of brown algae Natural polysaccharide	Biocompatible and biodegradable Adequate crosslinking capacity Relatively low cost Non-toxicity	Poor mechanical strength without crosslinking Low biological properties
**Synthetic polymer**	Poly(dimethylsiloxane) (PDMS) [50,51,52]	Silicon-based organic polymer	Non-toxic and inert Viscoelastic Homogeneous and isotropic	Lacks in bio-functional cues
	Polyvinyl alcohol (PVA) [53,54,55]	Water-soluble synthetic polymer	High solubility and biodegradability Relatively low cost Long-lasting durability High temperature stability	Lacks in bio-functional cues

**Table 2:** Advantages and disadvantages of conventional electrospinning and cell-electrospinning.

	Electrospinning	Cell-Electrospinning
**Advantages**	Simple process Provide controllable micro/nano-sized fibers Mimic the native ECM structure	All the same advantages of electrospinning High resolution (nanoscale) Efficient and fast nutrients/oxygen exchange Excellent cell-to-cell interaction Homogeneous cell distribution in strut
**Disadvantages**	Use of toxic solvents Insufficient cell infiltration Inhomogeneous cell distribution	Low mechanical properties Restrict to develop into 3D structure Low cell density controllability Low precision in fiber deposition

**Table 3:** Summary of various cells used in cell-electrospinning processes.

Cell Types	Solution	Reference
Osteoblast (MG63) cells	Alginate/poly(ethylene oxide) (PEO)/lecithin	[21]
Primary cardiomyocytes	Matrigel rich collagen biopolymer	[57]
C2C12 myoblast cells	Alginate/PEO	[30]
C2C12 myoblast cells	Fibrin/PEO	[58]
Primary porcine vascular smooth muscle cells (SMCs) and rabbit aorta SMCs	Poly(dimethyl siloxane) (PDMS)	[59,60]
PC-12 cells	Poly(l-lactic acid)	[61]
Human astrocytes (1321N1)	PDMS	[18,62]
Neuroblastoma (N2A) cells	Matrigel with high concentration of laminin	[20]
Adipose stem cells (ASCs)	Polyvinyl alcohol (PVA)	[22]
